# Differential Nutrient Use Efficiency and Biomass Partitioning of Interspecific Hybrids and Commercial Sugarcane Genotypes Under Early Drought Stress and Recovery Conditions

**DOI:** 10.3390/plants14243717

**Published:** 2025-12-05

**Authors:** Thanakorn Kulrat, Nakorn Jongrungklang, Sanun Jogloy, Nimitr Vorasoot, Anon Janket, Darunee Puangbut, Patcharin Songsri

**Affiliations:** 1 Department of Agronomy, Faculty of Agriculture, Khon Kaen University, Khon Kaen 40002, Thailand; 2 Northeast Thailand Cane and Sugar Research Center, Khon Kaen University, Khon Kaen 40002, Thailand; 3 Department of Agronomy, Faculty of Agriculture, Ubon Ratchatani University, Ubon Ratchatani 34190, Thailand; 4 Plant Production Technology, Faculty of Technology, Udon Thani Rajabhat University, Udon Thani 41000, Thailand

**Keywords:** *S. officinarum*, *S. spontaneum*, NUE, PUE, KUE, drought resistance, sugarcane breeding

## Abstract

Drought significantly impacts nutrient use efficiencies and sugarcane biomass. Interspecific hybridization between *Saccharum officinarum* and *Saccharum spontaneum* may improve drought resistance and enhance nutrient use efficiency. This research enhances the understanding of nutrient (N, P, and K) use efficiency and the biomass of interspecific hybrids and commercial sugarcane genotypes under early drought and recovery. The experiment was conducted using a split plot in a randomized complete block design (RCBD), with three replications. The main plot consisted of two water regimes (well-watered, WW, and early drought stress, DS), whereas the subplot consisted of six diverse sugarcane genotypes. Biomass, nitrogen use efficiency (NUE), phosphorus use efficiency (PUE), and potassium use efficiency (KUE) were measured 6, 8, and 12 months after transplanting (MAT). The results showed that drought reduced NUE, PUE, KUE, and biomass in all sugarcane genotypes throughout the drought period at 6 MAT. F03-362 and KK09-0358 had high biomass and NUE under drought stress. Meanwhile, F03-362 displayed consistently high biomass, NUE, PUE, and KUE during the recovery phase (8 MAT) as well as at 12 MAT under DS conditions, whereas TPJ04-768 showed high biomass only at 12 MAT. These contrasting responses highlight the important implications of selecting parental genotypes to improve nutrient use efficiency and biomass under early drought stress.

## 1. Introduction

Sugarcane is the primary raw material for sugar production, accounting for over 80% of global sugar production [1]. Additionally, the byproducts of sugar production, such as bagasse and molasses, can add significant value [2]. Sugarcane bagasse generates bioelectricity, and molasses can be used for ethanol production, a raw material for gasohol production. Sugarcane is vital to industry because it is not only a source of sugar but also a primary renewable energy source.

The most sugarcane plantation areas are in tropical and subtropical regions (such as Brazil, Thailand, and India). These countries frequently face drought stress due to erratic precipitation, which often impacts sugarcane yields [3,4]. Over 80% of Thailand’s sugarcane production is in rainfed areas, where droughts usually occur during the early growth stages [4,5]. Drought stress is an essential factor that restricts biomass and cane yield, causing reductions of over 50% in rainfed conditions [6]. Furthermore, drought stress affects the ability to take in nutrients, absorb nutrients efficiently, and utilize nutrients efficiently.

Nitrogen (N), phosphorus (P), and potassium (K) are crucial in sugarcane production, which is a heavy nutrient-feeding crop [7], incurring exorbitant costs for growers and becoming a serious issue for cane production [8]. Nevertheless, overuse of fertilizer could result in concerns with the environment such as soil deterioration and nutrient runoff [9]. Consideration of the efficiency of sugarcane use is therefore essential to finding a solution to this problem. However, nutrient use efficiency is usually limited by drought stress, which may be the important reason for yield reduction. Hoang et al. [10] reported that drought stress negatively impacts nutrition by limiting nitrogen uptake and reducing nitrogen usage efficiency, leading to decreased biomass. Enhancing nutrient use efficiency in sugarcane is a critical goal in cane production per area and minimizing nutrient applications of N, P, and K [11]. Enhancing its capacity to employ these inputs more efficiently can result in a number of advantages, including environmental health and sustainability [12]. However, the information on the effect of early drought on phosphorus and potassium use efficiency in sugarcane still needs to be documented. The information on the response to drought for nutrient efficiency was critical for drought-resistant breeding programs.

The moderately drought-tolerant sugarcane genotype was grown in almost all sugarcane crop areas, but it is insufficient when sugarcane is experiencing severe drought stress. Drought-tolerant sugarcane genotypes are hybrids derived from interspecific crossings between *S. officinarum* (commercial cane) and *S. spontaneum* (wild type) [5]. *S. spontaneum* has played an essential role in improving drought tolerance because *S. spontaneum* has a great root system that can increase water uptake and nutrient absorption ability where water is limited [13]. Mawan and Kaewpradit [14] revealed that *S. spontaneum* has a greater nitrogen absorption efficiency than commercial sugarcane genotypes because *S. spontaneum* has root exudates that can be used to improve biological nitrification inhibition by modifying the physical, chemical, or biological features of the rhizosphere, hence preventing nitrification in the soil, nutrient bioavailability, and plant development. In addition, *S. spontaneum* showed high nitrogen use efficiency under rainfed conditions [15]. However, nitrogen use efficiency (NUE) varied depending on the genetic variance of *S. spontaneum* [16]. Moreover, the previous study of Tippayawat et al. [17] reported that the interspecific hybrid genotypes showed greater drought tolerance than the commercial genotype in terms of biomass accumulation and physiological responses during drought periods (1 MAT–6 MAT), which mainly focused on physiological responses under drought conditions. However, the study of Tippayawat et al. [17] did not address the aspect of nutrient use efficiency, which is particularly interesting under drought stress. In addition to drought tolerance, these genotypes may also exhibit efficient nutrient use efficiency, which may be explained by the fact that they were produced from a cross with *S. spontaneum*. This observation prompted us to investigate the nutrient use efficiency of both interspecific hybrids and commercial sugarcane genotypes during the drought, recovery, and harvest stages. A previous study revealed that the commercial sugarcane genotypes considered as drought-tolerant had high nitrogen use efficiency under drought stress [10]. We raise the question of whether the interspecific hybrid sugarcane genotypes with drought tolerance may improve nutrient use efficiency. However, there is a lack of information on the nutrient use efficiency of interspecific hybrids compared to commercial sugarcane genotypes under early drought stress and recovery conditions.

The most interspecific hybrid sugarcane crosses, involving *S. officinarum* and *S. spontaneum*, offer genotypes suitable for breeding goals, including multifunctional cane (producing both sugar and energy) and improving drought resistance. Genotypes with high nutrient use efficiency may maintain robust growth and biomass accumulation while minimizing nutrient wastage, contributing to sustainable sugarcane production. Understanding the nutrient use efficiency of different sugarcane genotypes under early drought conditions is crucial for sugarcane breeding programs aimed at maintaining high biomass while ensuring efficient nutrient utilization. Thus, this study aimed to investigate the effect of early drought on the biomass and nutrient (N, P, and K) use efficiency of interspecific hybrids and commercial sugarcane.

## 2. Results

### 2.1. Weather, Soil Moisture, and Plant Water Status

**Figure 1:**
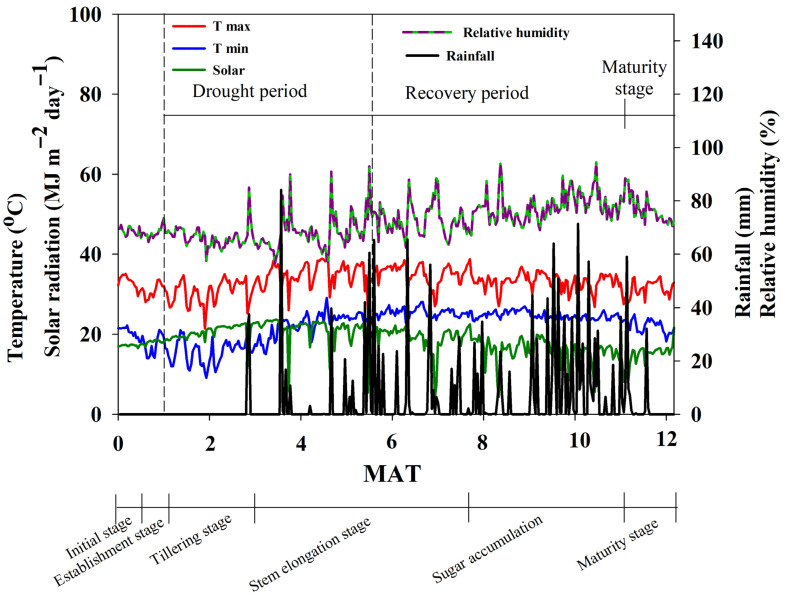
Maximum temperature (T max), minimum temperature (T min), solar radiation, rainfall, and relative humidity during the sugarcane crop growth period.

The means of minimum and maximum air temperatures were 22.1 and 33.2 °C, respectively (Figure 1), while the total amount of rainfall was 1207.2 mm. During the 5–6 MAT drought, rainfall was 165 mm. The daily relative humidity ranged from 56.5% to 94.5%, and the average solar radiation was 18.4 MJ m^−2^ day^−1^.

**Figure 2:**
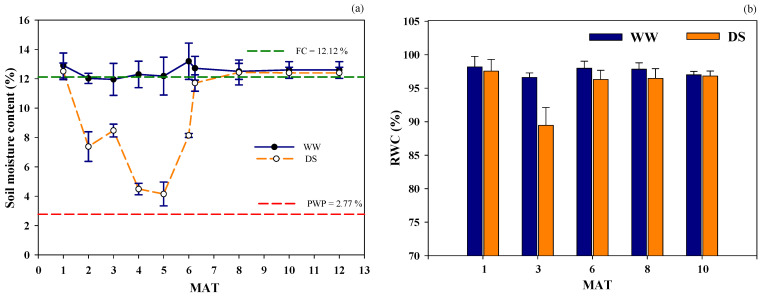
Soil moisture content (%) at soil depth of 0–60 cm (**a**), relative water content (%) (**b**) under well-watered (WW) and drought stress (DS) conditions during the crop growth period of sugarcane. Error bars represent standard errors of the means.

Soil moisture content was similar between well-watered (WW) and early drought stress (DS) conditions at 1 MAT because drought had not yet started in this period (Figure 2). However, the soil moisture content during the drought period at 4 MAT revealed clear distinctions between irrigated and drought treatments. Soil moisture decreased to 4.48% of drought treatment at 4 MAT, while soil moisture contents of DS increased by 8.1% due to rainfall at the end of the drought period (6 MAT). However, following the recovery beginning after 6 MAT, the values returned to field capacity at 187 days after transplanting (6 MAT + 1 week) and were maintained through 12 MAT. Relative water content (RWC) showed no significant difference between well-watered and drought treatments at 1 MAT. However, RWC differed significantly between stressed and non-stressed plants during the drought period. Drought reduced the RWC to 7.2% of WW at 3 MAT. Meanwhile, the RWC showed no difference between WW and DS treatments at 6 MAT because of rainfall. However, the RWC recovered to well-watered treatment levels during the recovery periods at 8 and 10 MAT.

### 2.2. Genotypic Variation and Genotype × Water Regime Interaction for Biomass and Nutrition Use Efficiency

The effects of genotype (G) and water regime (W) on biomass, total nitrogen uptake, absorption efficiency (NAE), and nitrogen use efficiency (NUE) were significant at 6, 8, and 12 MAT, but there were no significant differences among genotypes and between water regimes for nitrogen utilization (NUtE) at 6 and 8 MAT, respectively (Table S1). The W × G interaction effects were significant for biomass at 6 and 8 MAT, total nitrogen uptake and NAE at 12 MAT, NUtE at 8 and 12 MAT, and NUE at 12 MAT. Significant differences between water regimes (W) were observed for total phosphorus uptake, PAE and PUE, total potassium uptake, KAE, KUtE, and KUE at 6, 8, and 12 MAT except PUtE (Table S2). The difference in genotypes (G) was significant for all traits at 6, 8, and 12 MAT. The genotype by water regime (G × W) effect was substantial for PUE, KUtE, and KUE at 6 MAT, PUtE, potassium in the plant, KAE, and KUE at 8 MAT, and potassium in the plant, KAE, and KUtE at 12 MAT.

### 2.3. Responses of Biomass Production Under Well-Watered and Drought Stress Conditions

**Figure 3:**
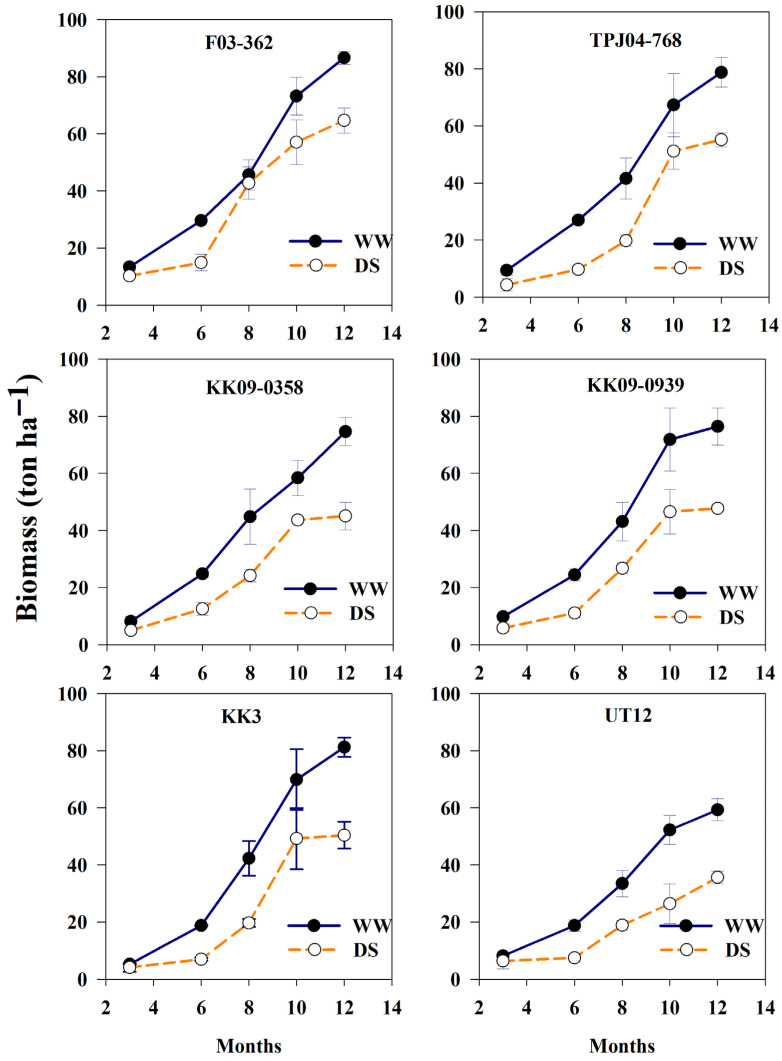
Biomass of six sugarcane genotypes under two water regimes (well-watered, WW, and drought stress, DS) with drought stress period (1–6 MAT) and recovery period (after 6–12 MAT) periods. Error bars represent standard errors of the means.

Early drought significantly reduced biomass production in all genotypes, with an average decrease of 43% compared with WW conditions at 6 MAT (Figure 3). Notably, F03-362 exhibited full biomass recovery to WW levels at 8 MAT. However, although all genotypes partially recovered after re-watering under DS conditions, none fully restored biomass to WW levels at 12 MAT. Under early drought stress, the six sugarcane genotypes showed two distinct response patterns. The first pattern, genotype, had continuously increased biomass to maturity stage (12 MAT), such as F03-362, TPJ04-768, and UT12. The second pattern shows a fast rise in biomass up to around 8–10 MAT, followed by a slower increase by 12 MAT, as seen in examples like KK09-0358, KK09-0939, and KK3.

### 2.4. Biomass Partitioning of Different Sugarcane Genotypes Under Well-Watered and Drought Conditions

**Figure 4:**
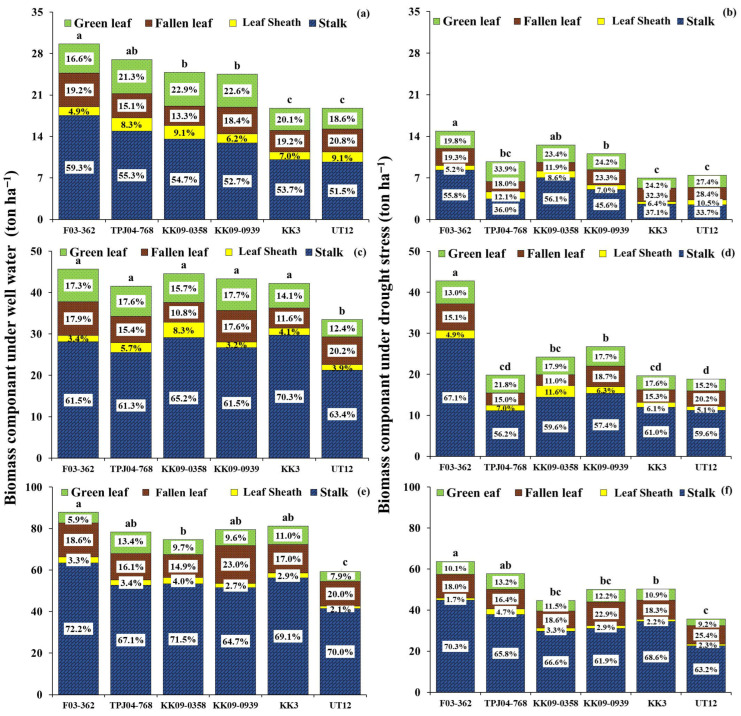
Biomass (stalk, leaf sheath, fallen leaves, and green leaves partitioning percentage) of 6 sugarcane genotypes under well-watered (WW) (left, (**a**,**c**,**e**)) and drought stress (DS) (right, (**b**,**d**,**f**)) conditions at 6, 8, 12 MAT. The same small letters were not significantly different by the least significant difference (LSD) at the *p* = 0.05 probability level.

Early drought dramatically decreased biomass proportions in the stalk, leaf sheath, fallen leaves, and green leaves in all sugarcane genotypes (Figure 4). However, different water regimes had a negligible impact on the alteration of the percentage of biomass in any part. The highest proportions of biomass were observed in the stalk, while the lowest was found in the leaf sheath under both water regimes at 6, 8, and 12 MAT. In addition, the increase in the percentage of biomass in stalk fractions depends on the growth stage and sugarcane genotypes. At stalk elongation, approximately 55% of biomass accumulates to the stalks (6 MAT), increases to more than 60% at 8 MAT, and then increases to a maximum value at crop maturity (12 MAT). Green leaves and leaf sheaths decreased with the age of the plant. The genotypes F03-362 and KK3 partitioned a higher fraction of final biomass to the stalk and smaller to leaves.

During the drought period at 6 MAT, early drought conditions resulted in an average reduction in total biomass by 56% compared to well-watered conditions (Figure 4). However, drought has not influenced the change in biomass partitioning in all sugarcane genotypes. The biomass accumulated more in the stalk than in the leaves under both conditions. The genotypes F03-362 and KK09-0358 preferred partitioning more than 50% of biomass to stalks under WW or DS conditions. Meanwhile, TPJ04-768, KK09-0939, KK3, and UT12 accumulated more in leaf organs than in the stalk under DS.

At 8 MAT, following re-watering after the early drought period (6 MAT), all genotypes exhibited partial adjustments in biomass allocation. Compared with the drought period, the dry weight of shoot organs—particularly green leaves and leaf sheaths—increased at 8 MAT under DS conditions, indicating a shift in biomass partitioning back toward shoot growth during the recovery phase. However, these increases were small and insufficient to restore biomass allocation to the levels observed under WW conditions. The total biomass of sugarcane genotypes subjected to DS was reduced compared to WW (Figure 4). Genotype F03-362 exhibited high total biomass, followed by KK09-0939 and KK09-0358, respectively, while UT12 showed the lowest biomass under drought conditions. The F03-362 exhibited excellent recovery, with the biomass value nearly reaching that of the control treatment, while other genotypes could not recover. Furthermore, the proportion of biomass to stalks was greater than that of leaves in all genotypes during this stage.

At 12 MAT, all sugarcane genotypes subjected to drought conditions significantly decreased in biomass (Figure 4). However, F03-362 and TPJ04-768 had low biomass reductions, while UT12 had a high reduction at the maturity stage. Interestingly, genotypes TPJ04-768 and KK3 recovered well from drought despite significant reductions throughout the drought period. The results indicated that the proportion of biomass to stalks was more significant than 60% for all sugarcane genotypes under drought conditions.

### 2.5. Plant Nutrient Content and Plant Nutrient Partitioning: Nitrogen, Phosphorus, and Potassium

#### 2.5.1. Nitrogen Content and Partitioning in Plant

**Figure 5:**
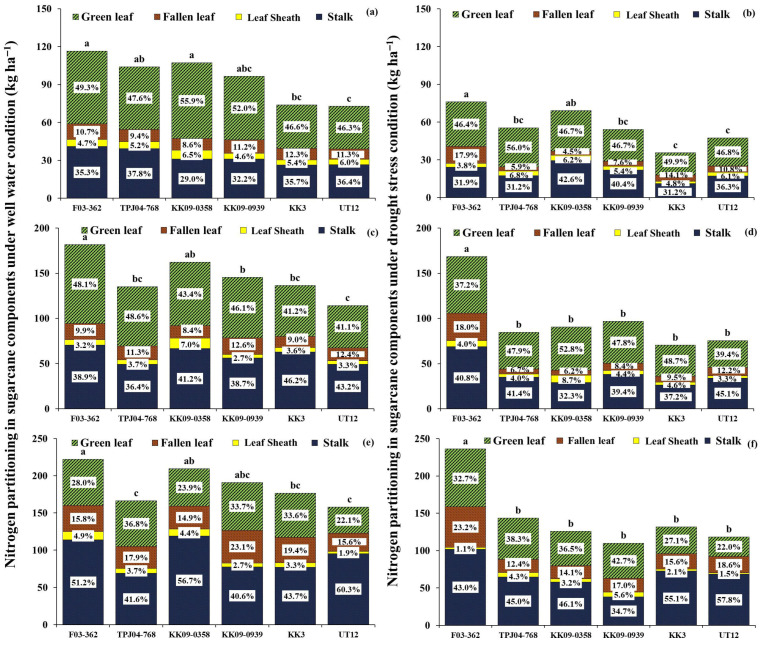
Nitrogen in plants (stalk, leaf sheath, fallen leaves, and green leaves partitioning percentage) of 6 sugarcane genotypes under well-watered (WW) (left, (**a**,**c**,**e**)) and drought stress (DS) (right, (**b**,**d**,**f**)) conditions at 6, 8, 12 MAT. The same small letters were not significantly different by the least significant difference (LSD) at the *p* = 0.05 probability level.

Drought adversely affected the N content at 6 MAT (Figure 5), decreasing 41% over WW. Two interspecific hybrids, F03-362 and KK09-0358, had the highest nitrogen content under WW and DS. Commercial sugarcane genotypes KK3 and UT12 showed the lowest N content. However, drought did not impact the nitrogen partitioning in the plant’s components in all sugarcane genotypes. The highest proportions of nitrogen content were observed in the green leaf following the stalk component, while the lowest was found in the leaf sheath under both water regimes. Interspecific hybrid KK09-0358 and F03-362 had a high percentage of nitrogen in green leaves (59.9 and 57.5 kg ha^−1^, respectively), while KK3 and UT12 showed a low percentage of nitrogen in green leaves (34.5 and 33.7 kg ha^−1^) at 6 MAT.

At 8 MAT, the N content could not recover to well-irrigated treatment following re-watering. F03-362 and KK09-0358 had the highest N content under fully irrigated (181.6 and 162.3 kg ha^−1^, respectively), while F03-362 had the highest N content (164.5 kg ha^−1^) under DS (Figure 5). Interestingly, F03-362 not only has a high nitrogen concentration but it also has slightly reduced N content under DS. Most sugarcane genotypes had the highest N levels in green leaves, except the F1 hybrid F03-362, which showed the greatest N allocation to the stalk.

At 12 MAT, the N content of stressed plants was reduced for all sugarcane genotypes, except for the F1 hybrid F03-362, which increased N content after recovery (Figure 5). Interestingly, the F1 hybrid F03-362 increased N content by an average of 6.3% compared with irrigated treatment after re-watering. In contrast, the N content of five sugarcane genotypes did not fully recover. The partitioning fraction to stalks increased at the maturity stage with a corresponding decrease in the fraction partitioned to leaves for all sugarcane genotypes except KK09-0939. In KK3 and UT12 genotypes, more than 50% of N content was partitioned to the stalk, whereas in F03-362, TPJ04-768, and KK09-0358, 45% of N content was partitioned to the stalk.

#### 2.5.2. Phosphorus Content and Partitioning in Plant

**Figure 6:**
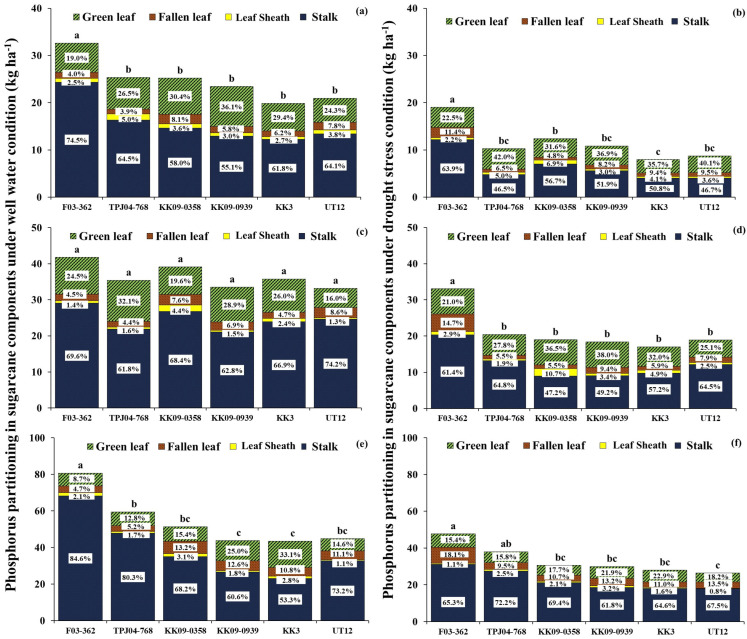
Phosphorus in plants (stalk, leaf sheath, fallen leaves, and green leaves partitioning percentage) of 6 sugarcane varieties under well-watered (WW) (left, (**a**,**c**,**e**)) and drought stress (DS) (right, (**b**,**d**,**f**)) conditions at 6, 8, 12 MAT. The same small letters were not significantly different by the least significant difference (LSD) at the *p* = 0.05 probability level.

Significant differences existed between water regimes regarding total P uptake, especially at 6 MAT. Drought reduced total phosphorus uptake compared to WW (53% for phosphorus uptake in plant reduction) (Figure 6). Sugarcane genotypes that experienced early drought did not fully recover in phosphorus value in plants after re-watering. The percentage of P in plant reduction was continuously reduced at 8 MAT (42% for P reduction) and 12 MAT (38% for P reduction). The water regimes did affect the phosphorus in the stalk ratio at 6 and 8 MAT; the P partitioning percentage of the stalk under WW was higher than in drought conditions. Specifically, the P partitioning percentage of green leaves increases in all drought conditions compared with WW at 6 MAT (drought period). The growth stage significantly affects P partitioning, with higher P values found in the stalk at later stages. On the other hand, when sugarcane was older, the P partitioning percentage in green leaves decreased.

At 6 MAT, the interspecific hybrid, F03-362 (F1), had the highest P content under both WW and drought conditions. However, almost all genotypes did not differ in P content under WW, while KK3 showed low P content under drought conditions. The highest proportions of P content were observed in the stalk (63.0% under WW and 52.8% under DS) following the green leaf, while the lowest was found in the leaf sheath under both water regimes. The F03-362 showed high stalk P levels in both conditions. At 8 MAT, sugarcane genotypes showed no significant difference in total P content under WW. On the other hand, P content was significantly higher among sugarcane genotypes under drought conditions. Phosphorus partitioning and stalks exhibited the highest percentage, followed by green leaves, fallen leaves, and leaf sheaths, with values of 56.6%, 31.9%, 6.8%, and 4.7%, respectively. F03-362 exhibited the highest total P content (33.0 kg ha^−1^) when compared to other genotypes; in addition, TPJ04-768, KK09-0358, KK09-0939, KK3, and UT12 showed no differences in total P content. F03-362 showed the highest P content in the stalk (20.3 kg ha^−1^) and fallen leaves (4.9 kg ha^−1^).

At 12 MAT under WW, sugarcane genotypes showed differences in the total P content. Stalk had the highest P partitioning percentage, followed by green leaves, fallen leaves, and leaf sheaths, with 70.0%, 18.3%, 9.6%, and 2.1%, respectively. In addition, F03-362 had the highest P in the stalk (68.3 kg ha^−1^), whereas KK3 had the lowest P in the stalk (23.2 kg ha^−1^). Thus, P content in the stalk had a high impact on promoting total P content, resulting in the F03-362 having the highest total P content. Under DS, F03-362 and TPJ04-768 showed a high total P content (47.7 and 38.1 kg ha^−1^), and UT12 had the lowest total P content (26.4 kg ha^−1^). Stalk had the highest P partitioning percentage, followed by green leaves, fallen leaves, and leaf sheaths, which had 66.8%, 18.6%, 12.7%, and 1.9%, respectively.

#### 2.5.3. Potassium Content and Partitioning in Plant

**Figure 7:**
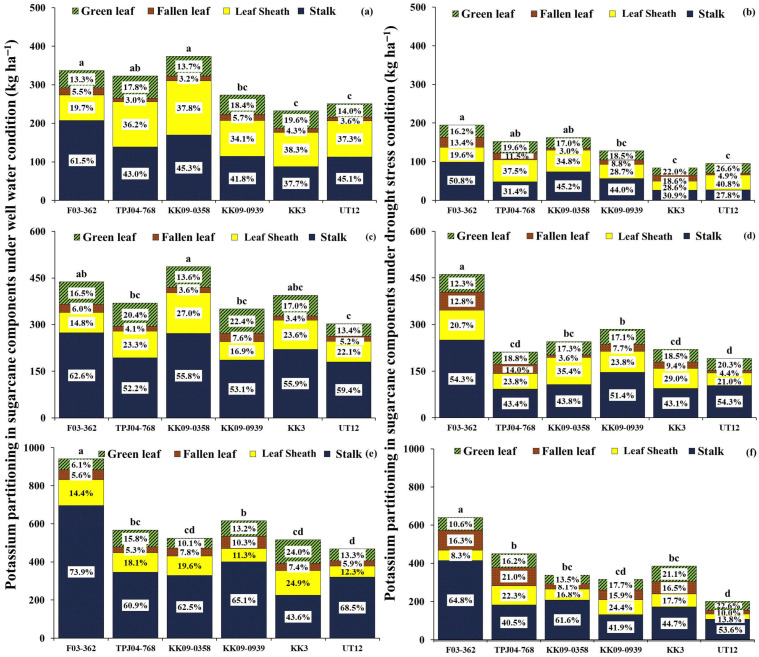
Potassium in plants (stalk, leaf sheath, fallen leaves, and green leaves partitioning percentage) of 6 sugarcane genotypes under well-watered (WW) (left, (**a**,**c**,**e**)) and drought stress (DS) (right, (**b**,**d**,**f**)) conditions at 6, 8, 12 MAT. The same small letters were not significantly different by the least significant difference (LSD) at the *p* = 0.05 probability level.

Significant differences between water regimes were observed for total K content, especially during drought stress at 6 MAT. Drought reduced total K uptake in plants compared to WW, with a loss of 54% (Figure 7). Sugarcane subjected to early drought stress did not fully recover its total K content after re-watering. The percentage of total K reduction in plants continued to decrease at 8 MAT (31%) and 12 MAT (36%). The water regimes did not affect the K in the plant component partitioning percentage. The impact of growth stages on the K partitioning revealed increased K values in the stalk and fallen leaves as the plant ages. On the other hand, the accumulation percentage of K in green leaves and leaf sheaths decreased with the increase in plant ages.

At 6 MAT under WW, differences in total K content were found among sugarcane genotypes. KK09-0358, F03-362, and TPJ04-768 exhibited a high total K content under both conditions. However, KK3 and UT12 showed low K content. Under WW conditions, the stem exhibited the highest percentage of K partitioning, at 45.8%, followed by the leaf sheath (33.9%), green leaves (16.1%), and fallen leaves (4.2%), respectively. In addition, K content in the stalk ranged from 87.6 to 207.4 kg ha^−1^; genotypes F03-362 and KK09-0358 showed the highest K content in the stalk, measuring 169.4 and 207.4 kg ha^−1^, respectively. Additionally, the K value in the leaf sheath ranged from 66.3 to 141.2 kg ha^−1^. Notably, KK09-0358 had the highest K value in the leaf sheath, measuring 141.2 kg ha^−1^.

Under DS, differences in the total K content among sugarcane genotypes were also observed, with values ranging from 83.7 to 194.9 kg ha^−1^. The F03-362, KK09-0939, and TPJ04-768 exhibited a high total K content, measuring 194.9, 162.3, and 152.4 kg ha^−1^, respectively. The stalk had the highest K partitioning percentage, 38.3%, followed by the leaf sheath, green leaves, and fallen leaves, with percentages of 31.7%, 20.0%, and 10.0%, respectively. Additionally, F03-362 exhibited the highest K value in the stalk (98.9 kg ha^−1^) and fallen leaves (26.2 kg ha^−1^). On the other hand, TPJ04-768 and KK09-0358 showed the highest K value in the leaf sheath, measuring 57.1 kg ha^−1^ and 56.5 kg ha^−1^, respectively.

At 8 MAT under WW, the partitioning of K content between the stalk and leaf sheath was significantly changed from 6 MAT; namely, the percentage of K partitioning in the leaf sheath was reduced; on the other hand, it was increased in the stalk. However, the total K content among sugarcane genotypes was different. The total K rate ranges from 302.5 to 486.5 kg ha^−1^. KK09-0358, F03-362, and KK3 had the highest total K uptake (486.5, 437.3, and 394.4 kg ha^−1^, respectively). Under DS, total K content ranged from 191.4 to 461.2 kg ha^−1^. F03-362 had the highest total K content (461.2 kg ha^−1^), while UT12 had the lowest total K uptake in plants (191.4 kg ha^−1^). Additionally, partitioning of K content between the stalk and leaf sheath was significantly changed from 6 MAT in almost all genotypes except for F03-362 and KK09-0358; these genotypes maintained the percentage of K partitioning in the leaf sheath; namely, it was not translocated from the leaf sheath to the stalk in F03-362 and KK09-0358.

Under WW at 12 MAT, a significant difference among sugarcane genotypes was observed for total K content. The partitioning of K content between stalk and trash (green leaves, fallen leaves, and leaf sheaths) significantly changed from 8 MAT. Stalk had the highest percentage of K partitioning on almost all genotypes except for KK3; KK3 showed less than 50% K partitioning percentage in stalk. However, the total K content among sugarcane genotypes was different. The total K rate ranged from 467.9 to 941.3 kg ha^−1^. The F03-362 had the highest total K content (941.3 kg ha^−1^). Under DS, total K content ranged from 200.5 to 640.2 kg ha^−1^. F03-362 had the highest total K content. However, the percentage of K content in the leaf sheaths of F03-362 and KK09-0358 was significantly reduced compared to 8 MAT. In contrast, other genotypes differed in K translocation percentage in the leaf sheath.

### 2.6. Nitrogen Use Efficiency

The NAE, NUtE, and NUE levels of sugarcane genotypes under DS were significantly lower than those under WW (Table 1). The reduction percentages ranged from 34.8 to 52.2%, 21.1 to 38.9%, and 49.4 to 64.0%, respectively.

At 6 MAT under WW, this study observed that NAE and NUtE are partially independent and contribute to NUE. The interspecific genotypes had a high NAE, resulting in high NUE, ranging from 175.1 to 211.5 g g^−1^. However, during DS, sugarcane genotypes differed significantly in NAE, NUtE, and NUE; NAE remains essential for promoting high NUE. F03-362 showed high NAE, NUtE, and NUE, and KK09-0358 exhibited high NAE and NUE.

At 8 MAT, under DS during the recovery period, NAE and NUE were significantly reduced compared to WW (34.1% and 40.0%, respectively) and significant among sugarcane genotypes for NAE, NUtE, and NUE. Under WW, the F03-362 and KK09-0358 had high NAEs of 1.30 and 1.16, respectively, and high NUEs of 326.4 and 319.1, respectively. KK3, TPJ04-768, and KK09-0939 exhibited a high NUtE of 316.3, 307.6, and 299.2, respectively, and a high NUE of 301.9, 296.9, and 309.7. Under DS, NUtE was not significant among sugarcane genotypes; NAE was different among sugarcane genotypes. In addition, the F03-362 genotype had the highest NAE (1.20) and the highest NUE (305.5 g g^−1^).

At 12 MAT, drought significantly reduced NAE, NUtE, and NUE by 25.1%, 17.27%, and 39.2%, respectively, compared to well-watered conditions. Under WW, F03-362, KK09-0358, and KK09-0939 showed high NAEs of 1.60, 1.50, and 1.36, and high NUEs of 634.1, 539.9, and 547.2 g g^−1^, respectively. However, genotypes TPJ04-768 and KK3 had a high NUtE (483.8 and 474.9) and high NUE of 568.7 and 597.8 g g^−1^, respectively. Under DS, the F03-362 had the highest NAE (1.7) and a high NUE of 473.6 g g^−1^.

### 2.7. Phosphorus Use Efficiency

Table 2 shows that sugarcane genotypes exposed to drought stress (DS) had much lower PAE and PUE than those under well-watered (WW) conditions. These reductions ranged from 41.6% to 59.7% for PAE and 49.4% to 64.0% for PUE.

At 6 MAT, there were differences in PAE and PUE among sugarcane genotypes under WW conditions. F03-362 showed the greatest values for PAE and PUE. Under DS, PAE and PUE were significant among sugarcane genotypes; F03-362 exhibited high PAE and PUE, while KK09-0358 showed high PUE. At 8 MAT, DS reduced PAE and PUE compared to WW for all sugarcane genotypes. The reduction among sugarcane varied from 20.7 to 52.4% and 8.8 to 56.4% for PAE and PUE, respectively. Under DS, sugarcane genotypes showed significance for PAE, PUtE, and PUE, in which F03-363 showed the highest PAE and PUE. At 12 MAT, DS significantly reduced PAE and PUE compared to WW. Among sugarcane genotypes, the reduction rate ranged from 31.4 to 41.1% and 25.3 to 39.9% for PAE and PUE, respectively. Under WW, sugarcane genotypes differed in PAE, PUtE, and PUE. Almost all genotypes showed higher PUE than UT12. However, F03-362 had a higher PAE than TPJ04-768, KK09-0358, KK09-0939, KK3, and UT12. However, the PUtE of KK09-0939 and KK3 had the highest values for PUtE. Under DS, PAE, and PUE showed significant differences among sugarcane genotypes; F03-362 had a high PAE and PUE. TPJ04-768 showed the highest PAE, while UT12 showed the lowest PAE and PUE.

Drought reduced PAE and PUE compared to WW. Although the plants recovered from drought stress, PAE and PUE showed less significant differences than in WW. When PAE and PUE responded to early drought stress, they did not fully recover. Under WW, almost all genotypes exhibited high PUE at harvest (12 MAT), except UT12. The classification of sugarcane genotypes to enhance the maintenance of PAE under DS was divided into two different patterns: (1) PAE exhibited high potential values (F03-362), and (2) PAE had low reduction values (TPJ04-768). Under DS, PAE substantially contributed to the high PUE of genetic variation.

### 2.8. Potassium Use Efficiency

At 6 MAT, drought reduced significantly in KAE and KUE compared to WW (Table 3), with a reduction rate of 49.4 to 64.0% and 49.4 to 64.0%, respectively. Under WW, sugarcane genotypes had different values for K uptake, KAE, and KUE, whereas F03-362 and TPJ04-768 had the highest KAE and KUE, and KK09-0358 had a high KAE. It was noted that KK3 and UT13 showed significantly low KAE and KUE. Under DS, KAE, KUtE, and KUE showed significant differences among sugarcane genotypes. F03-362 and KK09-0358 had high KAE and KUE; F03-362, KK09-0939, and KK3 had high KUtE.

After recovery at two months (8 MAT), DS had significantly lower KAE, KUtE, and KUE than WW. The average reductions in KAE, KUtE, and KUE were 31.2%, 12.9%, and 40%, respectively. Under WW, there were significant differences among sugarcane genotypes in KAE and KUE, with F03-362, KK09-0358, and KK3 having high KAE and KUE. Meanwhile, TPJ04-768 and KK09-0939 had high values for KUtE. On the other hand, UT12 had the lowest KAE and KUE. Under DS, KAE and KUE differed significantly across sugarcane genotypes, except for KUtE. F03-362 showed high KAE and KUE, while UT12 had the lowest for these parameters.

At 12 MAT and DS, significant reductions were observed in KAE and KUE compared to WW. These reductions ranged from 20.6% to 57.2% for KAE and 25.3% to 39.9% for KUE. Under WW, sugarcane genotypes were different for KUtE and KUE. Although F03-362 showed the lowest KUtE, it had the highest for KAE and a high value for KUE, the same as KK3. Under DS, there were significant differences between sugarcane genotypes regarding KAE, KUtE, and KUE. F03-362 had the highest KAE and KUE, while TPJ04-768 showed a higher KUE than other genotypes. It was noted that KK09-0939 and UT12 exhibited high KUtE under DS.

During the recovery period at 8 MAT, sugarcane performances showed differences compared to WW. Drought still exerted a lasting impact on KAE and KUE. Notably, at 12 MAT, KUtE fully recovered from early drought stress, while KAE and KUE did not fully recover. Low reductions in KAE and KUE of sugarcane genotypes can maintain KAE and KUE. Thus, F03-362 had the highest KUE, followed by TPJ04-768, and UT12 had the lowest KAE and KUE.

## 3. Discussion

Drought stress during the early growth stages, particularly during tillering and elongation phases, can significantly impact crop development and the yield of sugarcane. Early drought stress has drastically decreased shoot growth and biomass production [6,18]. Drought stress during the tillering stage inhibited leaf expansion and tiller formation, resulting in lower biomass productivity [19]. In addition, early drought may stunt root growth, leading to reduced water and nutrient uptake, further increasing stress, and compromising yield potential [20]. Reduced nutrient uptake and efficiency can directly impact biomass production in sugarcane [10,20]. The genotype demonstrates a notable capacity to maintain high nutrient uptake, and this nutrient efficiency may be vital for preserving substantial biomass during the initial phases of drought. During the recovery phase (8 MAT), the plants exhibit a subtle yet significant change, reflected in slight increases in the dry weight of the stalks, leaf sheaths, and lush green leaves. However, these improvements are insufficient for the biomass to return to the levels observed under optimal well-watered (WW) conditions. Furthermore, the adverse effects of drought stress may disrupt the delicate processes of biomass allocation and nutrient accumulation in various organs of the sugarcane plant. Gaining a comprehensive understanding of how drought-tolerant sugarcane genotypes manage their biomass and nutrient uptake is essential. This insight is invaluable for breeding programs focused on developing new sugarcane varieties that can not only endure water scarcity but also continue to thrive and maintain substantial biomass.

### 3.1. Biomass, Nutrient Accumulation, and Their Partitioning

The present study revealed that early drought stress reduced biomass production in both interspecific hybrids and commercial genotypes, consistent with previous findings by Mohanraj et al. [18], Leanasawat et al. [6], and Tippayawat et al. [17]. However, sugarcane hybrids derived through interspecific breeding generally display greater drought tolerance than commercial genotypes [6,18]. This increased resilience allows them to maintain better growth and biomass production even under WW. The findings showed that genotypes of interspecific sugarcane can maintain high biomass levels even in DS. Similarly, Leanasawat et al. [6] reported that interspecific sugarcane hybrids could continue producing a large amount of biomass even under DS. This trait is valuable in regions prone to water scarcity or erratic rainfall patterns, where crops must withstand extended periods of drought to ensure reliable yields. Therefore, the interspecific hybrids consistently exhibited better biomass than commercial genotypes during DS. The reduction in total biomass may be associated with a decline in the proportions of different organ biomass in the sugarcane plants.

Early drought dramatically decreased biomass proportions in the stalk, leaf sheath, fallen leaves, and green leaves in all sugarcane genotypes. Consequently, the reduction in dry weight in all plant organs could significantly affect the total amount of biomass produced in all sugarcane genotypes. Barbosa et al. [21] indicated that the reduction in the stalk and leaves significantly impacts the accumulation of total dry matter, which shows a high value in dry leaves and leaf sheath during drought stress in the tillering growth stage. Additionally, the results showed that, under both water regimes, the highest biomass values were observed in the stalk and the lowest in the leaf sheath at the elongation stage. Similarly, Singels et al. [22] found that biomass is allocated into stalks at a constant 70% of the biomass aboveground after stalk growth starts. Additionally, drought-tolerant sugarcane genotypes tend to allocate a higher proportion of biomass to stalks during drought conditions, suggesting an adaptive strategy to cope with water scarcity. According to Mohanraj et al. [18], drought-tolerant sugarcane hybrid genotypes exhibited a high stalk proportion. Specifically, the sugarcane genotypes F03-362 and KK09-0358 were found to have significant biomass accumulation in stalk organs during the drought period. This superior performance is consistent with the findings of Tippayawat et al. [17], who reported that these same genotypes were able to sustain higher photosynthetic activity under soil moisture deficit. These genotypes also exhibited reduced biomass under drought conditions, suggesting a potential adaptation to DS. Moreover, the ability of a genotype to effectively recover from drought and resume biomass production is essential for maintaining or increasing overall yield.

After the recovery period, each sugarcane genotype’s recovery ability significantly differed between commercial and interspecific hybrid sugarcane genotypes. Moreover, genotypes that capitalize on post-drought conditions to allocate resources effectively towards biomass production contribute considerably to overall yield potential. The interspecific hybrid sugarcane genotype F03-362 recovered biomass quickly, with its levels almost matching those seen under well-watered conditions. TPJ04-768 had high productivity during the late recovery period, which could help promote biomass accumulation after 8 MAT. Thus, the ability to recover after drought stress is a critical adaptation for the drought-tolerant sugarcane genotype. Khonghintaisong et al. [23] also reported that drought-tolerant sugarcane genotypes rapidly recovered for biomass after re-watering. Drought tolerance in the interspecific hybrids may also reflect genetic and biochemical traits inherited from *S. spontaneum*. Enhanced activation of stress-responsive genes and improved osmotic and antioxidant regulation—mechanisms previously associated with drought-tolerant sugarcane [15]—could partly explain the faster biomass recovery observed in F03-362.

This study noted that drought stress decreased the uptake of N, P, and K in plants by 41%, 53%, and 54%, respectively, when compared to WW. Similarly, Silva et al. [20] found that drought stress decreases the uptake of N and P in sugarcane genotypes. The observed phenomenon might be related to nutrient movement limitations within the soil, in which water deficit limits the plant root’s ability for nutrient uptake in the soil. The argument on nutrient uptake is supported by the mass flow mechanism, responsible for transporting N, P, and K nutrients from the soil to the roots. Drought stress restricts water movement in the soil, diminishing the mass flow rate and subsequently limiting nutrient availability around the roots of sugarcane plants. However, nutrient uptake capacity significantly differed among different sugarcane genotypes under drought and WW. According to Silva et al. [20], sugarcane genotypes usually differ regarding total nutrient uptakes under both conditions. In this study, the interspecific hybrid sugarcane genotypes F03-362 and KK09-0358 had high total nutrient uptake (N, P, K) in plants during drought imposed at 6 MAT, whereas KK3 and UT12 had a high reduction in nutrient uptake. Interspecific hybrid sugarcane genotypes tolerant to drought may exhibit higher nutrient uptake than commercial genotypes because they can maintain their nutrient uptake capacity. In addition, interspecific hybrid sugarcane genotypes exhibit low nutrient uptake reductions under drought stress. Moreover, this study observed that the genotypes F03-362 and KK09-0358 displayed high maintenance nutrient uptake in stalks and green leaves, resulting in high total nutrient uptake in plants under DS. Previous studies have noted that high root volumes and large root systems, traits potentially inherited from *S. spontaneum* [13], contribute to this enhanced nutrient uptake. Mawan and Kaewpradit [14] also reported that *S. spontaneum* had root exudates for improving biological nitrification inhibition for inhibiting nitrification in the soil nutrient bioavailability and plant growth by altering rhizosphere physical, chemical, or biological properties. This trait may be inherited by the interspecific hybrid genotypes with higher nutrient absorption efficiency than commercial genotypes. In this study, the interspecific hybrid genotypes are derived from a cross with *S. spontaneum*, which could explain why these interspecific genotypes exhibited higher drought tolerance than the commercial varieties.

At recovery, nutrient accumulation could not fully recover from DS even after re-watering. This could be due to fact that the the roots may take some time to recover from drought-induced damage fully, and then the root system may limit the plant’s ability to uptake nutrients efficiently [10]. Even after water availability is restored, nutrient uptake may remain limited, impacting overall nutrient accumulation in the plant [24]. However, the interspecific hybrids could maintain high nutrient uptake after drought ceased, such as genotypes F03-362 and TPJ04-767. Additionally, F03-362 exhibits good recovery for nutrient accumulation and high nutrient partitioning in leaf and stalk organs after the drought period. Since the plants produce more mass in the stalks, especially when the sugarcane is older, nutrients are stored in the stalks more [22]. During drought, sugarcane plants often redistribute nutrients from older tissues (leaves) to the stalks, where they are stored [25]. This increased biomass results in a more robust system for storing nutrients, which is especially critical under stress conditions like drought [20]. Therefore, F03-362, which was identified as a drought-resistant genotype with better biomass production, has higher nutrient storage volumes, especially in the stalks, than other genotypes, which supports the plant’s growth and recovery when favorable conditions return.

The percentage of nitrogen partitioning is high in green leaves, especially during stress relief, is well-founded, and highlights the importance of nitrogen allocation for plant recovery and physiological function restoration [26]. At the same time, P and K exhibit high partitioning in the stalk after recovery, underscoring their crucial roles in supporting cellular growth, energy transfer processes, and overall plant recovery [27,28,29]. After re-watering, P stored in the stalk can be mobilized and translocated to actively growing tissues, such as developing shoots and roots, to support ongoing growth and metabolic demands. This nutrient remobilization ensures the efficient utilization of P reserves and sustains plant growth and productivity after recovery [30]. Potassium supports stalk growth by playing a critical role in the effective transfer of water and nutrients during the stalk elongation stage [31,32]. However, the interspecific hybrid genotypes exhibited different nutrient uptakes than commercial sugarcane genotypes under recovery from droughts. Interestingly, after re-watering, the F03-362 genotypes had high nutrient uptake (N, P, and K). It was apparent that the F03-362 genotype exhibited the highest ability to uptake nutrients (N, P, and K) compared to other genotypes after the recovery period. The result suggested that F03-362 may possess favorable nutrient acquisition and utilization traits, highlighting its potential for improving nutrient use efficiency and productivity in sugarcane production.

However, the partitioning percentage of nutrients in different organs of sugarcane can vary depending on the plant growth stage. As sugarcane grows and develops, there are shifts in nutrient allocation among various plant organs to meet the changing metabolic demands and growth requirements. The growth stage of sugarcane impacted nutrient partitioning percentage and translocation [25,33]. Phosphorus showed different partitioning percentages in the sugarcane components compared to N and K. High P accumulation was maintained in the stalk during the stages of stalk elongation and maturity. In contrast, high N and K accumulation occurred in the leaves during the elongation stages and later translocated to the stalk during the mature stages.

The sugarcane genotypes showed high performance in nitrogen uptake, with the majority partitioned into green leaves (49.0%), followed by stalks (34.4%) and other organs (15.0%). This result indicated that nitrogen is essential for enhancing the growth and biomass accumulation of sugarcane during the elongation stage [33]. Potassium uptake was highly partitioned to the stalk, which may help by regulating osmotic adjustment, turgor pressure, and stomatal opening to reduce the impacts of water stress and promote continued development and elongation [25,34]. Phosphorus accumulation partitioned in the stalk tissues supports the high metabolic activity associated with sugarcane cell expansion, elongation, and structural development [30]. Additionally, stalk organs serve as major sinks for P storage and assimilation during recovery, with increased P accumulation supporting biomass accumulation, sucrose synthesis, and structural development in the stalk.

### 3.2. Nutrient Use Efficiency of Sugarcane Genotypes Under Drought and Recovery

The present study indicates that early drought stress can indeed lead to reduced NUE, PUE, and KUE in sugarcane plants. This could decrease biomass, as nutrients are essential for plant growth and biomass production.

Nitrogen is a fundamental nutrient that supports core metabolic processes, including photosynthetic activity, which collectively determine nitrogen use efficiency (NUE) [35,36,37,38]. In sugarcane, drought is known to impair these nitrogen-dependent processes [21,39], thereby reducing the plant’s capacity to utilize the available nitrogen effectively. This mechanistic understanding aligns with our results and previous findings showing that DS markedly decreased NUE [10] and, consequently, total biomass across all genotypes. Hoang et al. [10] stated that reduced NUE due to drought leads to decreased biomass. Additionally, reduced PUE caused by drought can seriously impact plant performance and biomass production by disrupting the metabolic processes, decreasing photosynthesis and energy production, and setting off stress responses [30]. In addition, drought stress significantly affects KUE in sugarcane, leading to drought-induced physiological limitations, reduced growth, and lower biomass production [40,41,42]. Potassium plays a key role in stomatal regulation, and disruption of this function under drought restricts transpiration and gas exchange, thereby limiting photosynthesis and carbon assimilation—processes essential for sustaining growth and biomass accumulation [40,41,42]. However, interspecific hybrid sugarcane genotypes, particularly F03-362 and KK09-0358, maintained high nutrient use efficiency during DS. These genotypes have adapted to improve nutrient absorption efficiency under drought conditions, as noted by Granato et al. [43], Hoang et al. [10], and Silva et al. [20]. Their adaptations may involve large and deep root systems capable of extracting water and nutrients from deeper soil layers, thereby minimizing drought’s detrimental impacts on biomass production [13,44]. Previous studies reported that the interspecific hybrid genotypes are derived from a cross with *S. spontaneum*, which has a large root system, which could increase the ability of water uptake and nutrient absorption under drought conditions [13].

At recovery, NUE, PUE, and KUE could not fully recover from DS even after re-watering in all sugarcane genotypes, suggesting that drought stress had a lasting impact on the ability of these plants to utilize nutrients efficiently. However, the interspecific hybrid sugarcane genotype F03-362 exhibited good NUE, PUE, and KUE recovery. The results revealed that the interspecific hybrid genotype contains genetic or adaptive traits to mitigate the adverse effects of drought stress on nutrient intake, promoting high biomass. The NUE, PUE, and KUE traits could be used as selection criteria for improving biomass under drought conditions.

The current study revealed that the interspecific F1 hybrid F03-362 genotype had high maintenance and good nutrient use efficiency recovery, leading to enhanced biomass production. Consequently, this genotype was identified as a drought-tolerant genotype. Additionally, this genotype can be used as a parent in sugarcane breeding to improve nutrient use efficiency and drought resistance.

## 4. Materials and Methods

### 4.1. Plant Materials and Experimental Design

Six sugarcane genotypes were studied from November 2020 to November 2021. Four sugarcane genotypes were interspecific hybrids of *S. officinarum* × *S. spontaneum* (F03-362 (F1) is drought-resistant [17]; TPJ04-768 (BC1), KK09-0358 (BC1), and KK09-0939 (BC2) and two commercial genotypes, including KK3 (adaptive) and UT12 (drought susceptible) [19]). The field experiment was carried out at the Khon Kaen Field Crops Research Center of Thaphra Station, Khon Kaen Province, Thailand (latitude 16°20′ N, longitude 102°4′ E, elevation 166.7 m above sea level). A split plot in a randomized complete block design (RCBD) with three replications was used. The main plot consisted of two regimes: well-watered (WW) and early drought stress (DS), where sugarcane was planted under early drought stress conditions (2–6 months after transplanting; MAT), and then there was full irrigation until harvest, and the subplot consisted of six sugarcane genotypes.

### 4.2. Soil Properties

Before planting, soil samples from 4 representative spots of each replication were collected using an auger at 0–30 and 30–60 cm depths to determine soil chemical and physical properties following the standard methods described by Zhang et al. [45]. The soil texture was sandy loam with a pH of 5.6 (averaged 0–60 cm). For sugarcane crops, the recommended pH range of the soil is between 5.5 and 6.5, which is also the best range for nutrient availability [46]. On average, at the depth of 0–60 cm, soil chemical and physical properties included total nitrogen (N) 0.015% (1530 kg ha^−1^) exchangeable potassium (K) 70.3 mg kg^−1^ (717 kg ha^−1^), exchangeable magnesium (Mg) 12.0 mg kg^−1^ (122 kg ha^−1^), organic matter (OM) 0.28%, electrical conductivity (EC) 0.016 dS m^−1^, exchangeable calcium (Ca) 118.0 mg kg^−1^ (1204 kg ha^−1^), available phosphorus (P) values 43.3 mg kg^−1^ (441 kg ha^−1^), bulk density was 1.7 g cm^−3^, 12.38% for field capacity (FC), and 2.04% for permanent wilting point (PWP).

### 4.3. Field and Crop Management

Land preparation was performed according to the standard procedures for experimental sugarcane fields: a 3-disk and a 7-disk tractor were used to plow, followed by harrowing. The plant holes were dug manually to a diameter of 20 cm and a depth of 20 cm. One-eye-bud sets of each sugarcane genotype were planted in separate plastic bags to promote higher homogeneity of the plants. After three weeks, the uniformity plants (at the 3-leaf stage) were transplanted in the field. The subplot consisted of eight rows, each 13 m long, with a 1.5 m row spacing and a 0.5 m plant spacing. Uniform-size plantlets were transplanted into the holes by covering them with soil until 10 cm below the soil surface.

Fertilizers were applied based on the soil analysis results and the recommendations for sugarcane fertilizer use [47]. N-P_2_O_5_-K_2_O fertilizer was applied at 137.5 kg ha^−1^, 31.3 kg ha^−1^, and 100 kg ha^−1^, respectively. In addition, N and K were split and applied two times, first at 1 MAT and then at 5 MAT, while P fertilizer was applied as a basal dose at 1 MAT.

Weeds were controlled with a chloroacetanilide herbicide (Alachlor 48% *w*/*v* EC: 2-chloro-2′, 6′-diethyl-/V-methoxymethy) applied at pre-emergence, and hand weeding was performed as necessary.

### 4.4. Water Regimes Management

A mini-sprinkler ensured uniform soil moisture before water treatment (0–30 days post-transplanting), and drip irrigation systems were installed during the water treatment periods. The mini-sprinklers were set up with lines spaced 4 meters apart and sprinkler heads also placed every 4 meters. A surface drip irrigation system was installed at a spacing of 0.5 m between drip lines and 0.5 m between emitters. During crop establishment, water was applied evenly across the experimental field using a mini-sprinkler system from transplanting until one month after transplanting (MAT). After this period, a drip irrigation system was implemented. After 1 MAT, early drought treatment was imposed by withholding water until 6 MAT (5 months for water withholding), and after 6 MAT, the plant was re-watered at FC and maintained at this level until harvest. The well-watered treatment was maintained at FC throughout the crop growth cycle. The total amount of water applied was calculated according to crop water requirement as described by Khonghintaisong et al. [19].

ET crop = ETo × Kc(1)

where ET crop is the crop water requirement (mm day^−1^); ETo is the evapotranspiration of a reference crop under a specified condition calculated by the evaporation pan method (ETo = Kp × Epan; Kp = pan coefficient (class A plan with green fetch) and Epan = pan evaporation (mm day^−1^)); Kc is the crop water requirement coefficient for sugarcane (initial stage (0–20 days after planting; DAP, Kc = 0.55), establishment stage (21–36 DAP, Kc = 0.80), tillering stage (37–96 DAP, Kc = 0.90), stem elongation stage (97–236 DAP, Kc = 1.10), yield formulation stage or sugar accumulation (237–336 DAP, Kc = 1.05), and maturity (337–365 DAP, Kc = 0.60)) [19].

The daily irrigation requirement was calculated according to the crop water demand, which was determined from Epan. Irrigation was scheduled at 2–3 day intervals, with the water amount adjusted for each event to maintain soil moisture within ±1% of field capacity. The irrigation frequency and amount were adjusted in response to rainfall events to prevent excessive soil moisture and to ensure precise maintenance of the WW treatment.

### 4.5. Data Collection

#### 4.5.1. Meteorological Data and Soil Moisture Content

The weather data, including rainfall (mm), pan evaporation (mm), solar radiation (MJ m^−2^ day^−1^), relative humidity (%), and minimum–maximum temperatures (°C), were recorded throughout the experiment period from a weather station located at 100 m from the experimental site. The soil moisture content (SMC) was measured at one-month intervals during drought stress periods (1, 2, 3, 4, 5, and 6 MAT), and then recovery periods were measured at 187 days after transplanting (6 MAT + 1 week) and at 8, 10, and 12 MAT by gravimetric method at depths of 0 to 60 cm. The soil samples were oven-dried at 105 °C for 72 h or until the weight was consistent. The percentage of soil moisture content was calculated as follows.

SMC (%) = [(wet soil mass − dry soil mass)/dry soil mass] × 100(2)

#### 4.5.2. Relative Water Contents

Relative water content (RWC) was evaluated at 1, 3, 6, 8, and 10 MAT from the bottom, middle, and top positions of the fully expanded leaf. For each sampling time, the leaf was cut into 2 cm^2^ at 9:00 to 11:00 a.m., put into a tube, and stored immediately in an icebox. After recording their fresh weight, samples were soaked in distilled water for 24 h, then surface-dried to measure their saturated weight. The weight of the water-saturated leaf was also calculated. After 48 h of oven drying at 80 °C, the leaf samples were weighed after drying. RWC was determined using the following method from Equation [48].

RWC (%) = [(leaf fresh weight − leaf dry weight)/(leaf saturated weight − leaf dry weight)] × 100(3)

#### 4.5.3. Biomass and Nutrient Content Determinations

Three stools in each sub-plot (area of 2.25 m^2^) were randomly selected from the middle row at 3, 6, 8, 10, and 12 MAT to determine biomass. Samples for nutrient (N, P, and K) analysis were collected at 6, 8, and 12 MAT from three stools per sub-plot. The plants were divided into stalks, fallen leaves, leaf sheath, and green leaves after being cut at the soil surface. The weight was then determined by weighing the fresh weight of each part. A subsample of 10% from each plant organ was collected after the total fresh weight was noted to calculate the dry weight. The dry weight of each sample was recorded after oven-drying at 70 °C for 72 h or until they reached a consistent weight. The total biomass was obtained by adding the weights of all plant organs. The dry weight values were converted and expressed in kilograms per square meter.

After drying, the samples were further ground in an electric stainless-steel blender. A 0.5 g sample was digested by the Kjeldahl digestion method for the N analysis and the wet oxidation method for the determination of P and K. An Auto Analyzer 3 (Germany’s Auto Analyzer 3) was used to measure the N content, according to the method described by Sáez-Plaza et al. [49]. Phosphorus was measured by spectrophotometer (U-2900 UV/VIS, Hitachi, Tokyo, Japan) according to the method described by Aboyeji et al. [50]. A flame photometer (M410 flame photometer, Sherwood Scientific, Cambridge, UK) was used to determine the content of K.

#### 4.5.4. Nutrient Use Efficiency

Total N, P, and K uptake in plants was calculated for each element by multiplying the nutrient concentration in each plant organ (stalk, leaf sheath, fallen leaves, and green leaves) by its dry weight (kg ha^−1^), and summing over all organs. The results are expressed in kg ha^−1^. Nutrient (N, P, and K) absorption efficiency (NAE, PAE, and KAE) was calculated as total nutrient (N, P, and K) uptake (kg ha^−1^) divided by the amount of fertilizer applied (kg ha^−1^). Nutrient (N, P, and K) utilization efficiency (NUtE, PUtE, and KUtE) was calculated as total plant dry weight (kg ha^−1^) divided by total nutrient (N, P, and K) uptake (kg ha^−1^). Nutrient (N, P, and K) use efficiency (NUE, PUE, and KUE) for biomass was calculated as the product of nutrient (N, P, and K) absorption efficiency and nutrient (N, P, and K) utilization efficiency. For clarity, “nutrient applied” refers to the fertilizer applied in the experiment and does not include soil nutrient content [43].

Nitrogen utilization efficiency (NUtE (g g^−1^)) = total dry weight/total nitrogen uptake (TNU) (4)

Nitrogen absorption efficiency (NAE) = TNU/N applied(5)

Nitrogen use efficiency (NUE (g g^−1^)) = NUtE × NAE (6)

Phosphorus utilization efficiency (PUtE (g g^−1^)) = total dry weight/total phosphorus uptake (TPU) (7)

Phosphorus absorption efficiency (PAE) = TPU/P applied(8)

Phosphorus use efficiency (PUE (g g^−1^)) = PUtE × PAE (9)

Potassium utilization efficiency (KUtE (g g^−1^)) = total dry weight/total potassium uptake (TKU) (10)

Potassium absorption efficiency KAE = TKU/K applied(11)

Potassium use efficiency (KUE (g g^−1^)) = KUtE × KAE (12)

### 4.6. Statistical Analysis

Analysis of variance in a split plot design was performed for each character [51]. Differences between means were tested by least significant difference (LSD) at an alpha level 0.05. All calculation procedures used Statistix 10 (Analytical Software, Tallahassee, FL, USA, 2013).

## 5. Conclusions

Early drought significantly reduced total biomass, nutrient uptake, absorption, and nutrient use efficiency (N, P, and K) in interspecific hybrid and commercial sugarcane genotypes. However, considering that the interspecific hybrid genotypes were more resistant to drought, they would have maintained high nutrient uptake and nutrient use efficiency and provided high biomass under drought conditions. Drought did not influence the change in biomass and nutrient partitioning in all sugarcane genotypes, but it varied with the growth stage. High biomass and nutrient partitioning (N, P, and K) accumulation was observed in the stalk organ at the maturity stage. After recovery, the mean values of all observed traits increased for interspecific hybrid genotypes, yet they were not completed under WW. Interspecific hybrid genotypes had low reduction and high recovery efficiency under DS. Notably, interspecific hybrid genotype F03-362 exhibited excellent performance in high biomass potential under WW coupled with maintained high nutrient uptake and nutrient use efficiency during drought stress and recovery periods under DS. The results indicated that the sugarcane genotype had high nutrient uptake and nutrient use efficiency; good recovery in these traits could be attributed to high biomass. This genotype could be used as a parent in the sugarcane breeding programs to improve nutrient use efficiency and enhance biomass under early drought stress.

## Figures and Tables

**Table 1:** Nitrogen absorption efficiency (NAE), nitrogen utilization efficiency (NUtE), and nitrogen use efficiency (NUE) under well-watered and drought stress conditions of 6 sugarcane genotypes.

Genotype	NAE				NUtE				NUE			
	WW		DS		WW		DS		WW		DS	
6 MAT												
F03-362	0.83	a	0.54	a	256.8		198.8	a	211.5	a	106.5	a
TPJ04-768	0.74	a	0.40	bc	263.2		175.2	c	192.9	a	69.4	bc
KK09-0358	0.77	a	0.49	ab	232.2		183.1	bc	177.3	a	89.7	ab
KK09-0939	0.69	ab	0.39	bc	264.0		204.9	a	175.1	a	79.0	b
KK3	0.53	b	0.25	d	254.3		195.9	ab	134.3	b	49.8	c
UT12	0.52	b	0.34	cd	258.9		158.3	d	134.1	b	53.3	c
Mean	0.7 A		0.4 B		254.9 A		186.0 B		170.9 A		74.6 B	
F-test	*		**		ns		**		**		**	
C.V. (%)	17.1		17.9		13.1		4.4		12.2		16.7	
8 MAT												
F03-362	1.30	a	1.20	a	252.3	c	257.5		326.4	a	305.5	a
TPJ04-768	0.97	bc	0.61	b	307.6	ab	233.9		296.9	a	141.2	c
KK09-0358	1.16	ab	0.65	b	275.2	bc	267.3		319.1	a	173.1	bc
KK09-0939	1.04	b	0.69	b	299.2	ab	281.1		309.7	a	194.9	b
KK3	0.97	bc	0.50	b	316.3	a	281.3		301.9	a	140.5	c
UT12	0.82	c	0.54	b	295.8	ab	254.0		243.4	b	134.8	c
Mean	1.0 A		0.7 B		291.1 A		262.5 A		299.6 A		181.7 B	
F-test	**		**		*		ns		*		**	
C.V. (%)	10.4		17.1		7.0		7.4		8.8		14.6	
12 MAT												
F03-362	1.59	a	1.69	a	400.0	b	282.1	d	634.1	a	473.6	a
TPJ04-768	1.19	c	1.03	b	483.8	a	386.8	ab	568.7	a	394.0	b
KK09-0358	1.50	ab	0.90	b	358.1	b	359.3	bc	539.9	a	322.2	c
KK09-0939	1.36	abc	0.79	b	401.9	b	434.3	a	547.2	a	341.2	c
KK3	1.26	bc	0.94	b	474.9	a	385.6	ab	597.8	a	359.9	bc
UT12	1.13	c	0.85	b	379.2	b	313.7	cd	426.4	b	259.1	d
Mean	1.3 A		1.0 B		416.3 A		360.3 B		552.3 A		358.3 B	
F-test	**		**		**		**		**		**	
C.V. (%)	9.8		14.9		9.1		9.6		9.8		7.6	

Means followed by the same small letter in a column and means followed by the same capital letter in each row were not significantly different by least significant difference (LSD) at *p* = 0.05 probability level. * Significant at *p* ≤ 0.05, ** significant at *p* ≤ 0.01 and ns = non significance.

**Table 2:** Phosphorus absorption efficiency (PAE), phosphorus utilization efficiency (PUtE), and phosphorus use efficiency (PUE) under well-watered and drought stress conditions of 6 sugarcane genotypes.

Genotype	PAE				PUtE				PUE			
	WW		DS		WW		DS		WW		DS	
	6 MAT											
F03-362	2.0	a	1.2	a	929.5		781.0		1835.7	a	924.0	a
TPJ04-768	1.6	b	0.6	bc	1084.2		950.7		1674.3	ab	602.4	bc
KK09-0358	1.6	b	0.8	b	991.7		1028.7		1538.7	b	778.4	ab
KK09-0939	1.5	b	0.7	bc	1076.0		1085.8		1519.4	b	685.7	b
KK3	1.2	b	0.5	c	977.8		875.1		1165.3	c	432.1	c
UT12	1.3	b	0.5	bc	893.7		852.1		1163.6	c	462.4	c
Mean	1.5 A		0.7 B		992.1 A		928.9 A		1482.8 A		647.5 B	
F-test	**		**		ns		ns		**		**	
C.V. (%)	14.6		20.4		15.4		15.2		6.1		16.7	
	8 MAT											
F03-362	2.6		2.1	a	1139.7		1295.4	b	2905.7		2651.3	a
TPJ04-768	2.2		1.3	b	1180.6		983.1	c	2603.4		1225.7	c
KK09-0358	2.4		1.2	b	1168.8		1282.3	b	2839.9		1501.9	bc
KK09-0939	2.1		1.1	b	1325.2		1583.5	a	2706.8		1774.2	b
KK3	2.2		1.1	b	1274.1		1155.0	bc	2795.7		1219.1	c
UT12	2.1		1.2	b	1031.1		1007.6	c	2092.0		1170.3	c
Mean	2.3 A		1.3 B		1186.6 A		1217.8 A		2657.3 A		1590.4 B	
F-test	ns		**		ns		**		ns		**	
C.V. (%)	16.5		19.6		11.6		9.2		22.2		18.8	
	12 MAT											
F03-362	5.0	a	3.0	a	1092.0	d	1373.4		5372.1	a	4011.0	a
TPJ04-768	3.7	b	2.4	ab	1515.3	bc	1488.4		5600.7	a	3419.1	b
KK09-0358	3.2	bc	1.9	bc	1457.6	bcd	1502.3		4627.4	ab	2838.7	c
KK09-0939	2.7	c	1.9	bc	1797.2	ab	1596.5		4799.1	a	2961.3	bc
KK3	2.7	c	1.7	bc	1913.6	a	1835.1		5036.8	a	3123.5	bc
UT12	2.8	bc	1.6	c	1327.7	cd	1354.5		3677.1	b	2209.3	d
Mean	3.3 A		2.1 B		1517.2 A		1525.0 A		4852.2 A		3093.8 B	
F-test	**		**		**		ns		**		**	
C.V. (%)	15.2		18.0		14.4		12.8		11.3		9.2	

Means followed by the same small letter in a column and means followed by the same capital letter in each row were not significantly different by least significant difference (LSD) at *p* = 0.05 probability level. ** Significant at *p* ≤ 0.01 and ns = non significance.

**Table 3:** Potassium absorption efficiency (KAE), potassium utilization efficiency (KUtE), and potassium use efficiency (KUE) under well-watered and drought stress conditions of 6 sugarcane genotypes.

Genotype	KAE				KUtE				KUE			
	WW		DS		WW		DS		WW		DS	
	6 MAT											
F03-362	2.4	a	1.4	a	256.8		198.8	a	214.0	a	107.7	a
TPJ04-768	2.3	ab	1.1	ab	263.2		175.2	c	195.2	ab	70.2	bc
KK09-0358	2.7	a	1.2	ab	232.2		183.1	bc	179.4	b	90.8	ab
KK09-0939	2.0	bc	0.9	bc	264.0		204.9	a	177.1	b	79.9	b
KK3	1.7	c	0.6	c	254.3		195.9	ab	135.9	c	50.4	c
UT12	1.8	c	0.7	c	258.9		158.3	d	135.7	c	53.9	c
Mean	2.2 A		1.0 B		254.9 A		186.0 A		172.9 A		75.5 B	
F-test	**		**		ns		**		**		**	
C.V. (%)	10.4		18.7		13.1		4.4		6.1		16.7	
	8 MAT											
F03-362	3.2	ab	3.3	a	104.6		92.5		330.2	a	309.1	a
TPJ04-768	2.7	bc	1.5	cd	113.4		93.3		300.4	a	142.9	cd
KK09-0358	3.5	a	1.8	bc	92.9		100.2		322.6	a	175.1	bc
KK09-0939	2.5	bc	2.1	b	127.7		94.2		313.4	a	193.6	b
KK3	2.9	abc	1.6	cd	107.6		89.6		305.5	a	142.1	cd
UT12	2.2	c	1.4	d	114.0		99.3		241.9	b	136.4	d
Mean	2.8 A		1.9 B		110.0 A		94.8 B		302.3 A		183.2 B	
F-test	*		**		ns		ns		**		**	
C.V. (%)	13.7		8.5		17.8		11.0		5.8		11.2	
	12 MAT											
F03-362	6.8	a	4.6	a	92.1	c	102.4	c	626	a	467.6	a
TPJ04-768	4.1	bc	3.3	b	139.7	ab	125.1	bc	569	b	398.6	b
KK09-0358	3.8	cd	2.5	bc	142.7	ab	133.3	bc	539	b	326.0	c
KK09-0939	4.5	b	2.3	cd	124.1	b	151.4	ab	553	b	345.2	c
KK3	3.7	cd	2.8	bc	159.1	a	132.7	bc	587	ab	364.2	bc
UT12	3.4	d	1.4	d	127.1	b	180.8	a	429	c	257.6	d
Mean	4.4 A		2.8 B		130.8 A		137.6 A		550.6 A		359.9 B	
F-test	**		**		**		**		**		**	
C.V. (%)	8.0		17.2		10.8		14.6		5.5		7.1	

Means followed by the same small letter in a column and means followed by the same capital letter in each row were not significantly different by least significant difference (LSD) at *p* = 0.05 probability level. * Significant at *p* ≤ 0.05, ** significant at *p* ≤ 0.01 and ns = non significance.

## Data Availability

Data are contained within the article and Supplementary Materials.

## Supplementary Materials

The following supporting information can be downloaded at https://www.mdpi.com/article/10.3390/plants14243717/s1, Table S1: Mean squares from ANOVA for biomass, total nitrogen uptake, nitrogen absorption (NAE), nitrogen utilization efficiency (NUtE), and nitrogen use efficiency (NUE) at 6, 8, and 12 MAT under two water regimes of six sugarcane genotypes. Table S2: Mean squares from ANOVA for total phosphorus uptake, phosphorus absorption (PAE), phosphorus utilization efficiency (PUtE), phosphorus efficiency (PUE), potassium in the plant, potassium absorption (KAE), potassium utilization efficiency (KUtE), and potassium use efficiency (PUE) at 6, 8, and 12 MAT under two water regimes of six sugarcane genotypes.

## Abbreviations

The following abbreviations are used in this manuscript:

DAP	days after planting
DS	early drought stress
KAE	potassium absorption efficiency
KUE	potassium use efficiency
KUtE	potassium utilization efficiency
MAT	months after transplanting
NAE	nitrogen absorption efficiency
NUE	nitrogen use efficiency
NUtE	nitrogen utilization efficiency
PAE	phosphorus absorption efficiency
PUtE	phosphorus utilization efficiency
PUE	phosphorus use efficiency
RWC	relative water content
SMC	soil moisture content
WW	well-watered

## References

[1] Lee H., Sohn Y.J., Jeon S., Yang H., Son J., Kim Y.J., Park S.J. (2023). Sugarcane wastes as microbial feedstocks: A review of the biorefinery framework from resource recovery to production of value-added products. Bioresour. Technol..

[2] Wani A.K., Rahayu F., Fauziah L., Suhara C. (2023). Advances in safe processing of sugarcane and bagasse for the generation of biofuels and bioactive compounds. J. Agric. Food Res..

[3] Zhao D., Li Y.R. (2015). Climate change and sugarcane production: Potential impact and mitigation strategies. Int. J. Agron..

[4] Pipitpukdee S., Attavanich W., Bejranonda S. (2020). Climate change impacts on sugarcane production in Thailand. Atmosphere.

[5] Khumla N., Sakuanrungsirikul S., Punpee P., Hamarn T., Chaisan T., Soulard L., Songsri P. (2022). Sugarcane breeding, germplasm development and supporting genetics research in Thailand. Sugar Tech..

[6] Leanasawat N., Kosittrakun M., Lontom W., Songsri P. (2021). Physiological and agronomic traits of certain sugarcane genotypes grown under field conditions as influenced by early drought stress. Agronomy.

[7] Hisbani W.A., Manono B.O., Nawaz M., Mehmood K., Hasnain Z., Rais A., Irshadz S., Ibrar D., Siddiqui M.H., Alamri S. (2025). Assessing the influence of potassium fertilizer variations on growth, yield, and crop quality of sugarcane (*Saccharum officinarum* L.) genotypes. Int. J. Plant Prod..

[8] Rossetto R., Dias F.L.F., Landell M.G.A., Cantarella H., Vitti A.C., Perecin D. (2010). N and K fertilisation of sugarcane ratoons harvested without burning. Proc. Int. Soc. Sugar Cane Technol..

[9] Bashir M.T., Ali S., Ghauri M., Adris A., Harun R. (2013). Impact of excessive nitrogen fertilizers on the environment and associated mitigation strategies. Asian J. Microbiol. Biotechnol. Environ. Sci..

[10] Hoang D.T., Hiroo T., Yoshinobu K. (2019). Nitrogen use efficiency and drought tolerant ability of various sugarcane varieties under drought stress at early growth stage. Plant Prod. Sci..

[11] Hawkesford M.J. (2012). Improving nutrient use efficiency in crops. Encyclopedia of Life Sciences.

[12] Kumar B., Sinha S.K., Kumar A., Kumari A. (2024). Exploring the impact of organic-inorganic coupling on nutrient use efficiency and cane yield in calcareous soils of the Indo-gangetic Plains of India. J. Adv. Biol. Biotechnol..

[13] Cheavegatti-Gianotto A., De Abreu H.M.C., Arruda P., Bespalhok Filho J.C., Burnquist W.L., Creste S., Di Ciero L., Ferro J.A., Figueira A.V.D.O., Filgueiras T.D.S. (2011). Sugarcane (*Saccharum* X *officinarum*): A reference study for the regulation of genetically modified cultivars in Brazil. Trop. Plant Biol..

[14] Mawan N., Kaewpradit W. (2022). Sugarcane root exudate impact on the potential nitrification rate and N dynamics in the rhizosphere. Rhizosphere.

[15] Luo T., Li C.N., Yan R., Huang K., Li Y.R., Liu X.Y., Lakshmanan P. (2023). Physiological and molecular insights into the resilience of biological nitrogen fixation to applied nitrogen in *Saccharum spontaneum*, wild progenitor of sugarcane. Front. Plant Sci..

[16] Mohanraj K., Manjunatha T., Mahadevaiah C., Suganya A., Adhini S.P., Geetha P. (2020). Genetic variability for nitrogen use efficiency in interspecific and intergeneric hybrids of sugarcane. Int. J. Curr. Microbiol. App. Sci..

[17] Tippayawat A., Jogloy S., Vorasoot N., Songsri P., Kimbeng C.A., Jifon J.L., Janket A., Thangthong N., Jongrungklang N. (2023). Differential physiological responses to different drought durations among a diverse set of sugarcane genotypes. Agronomy.

[18] Mohanraj K., Hemaprabha G., Vasantha S. (2021). Biomass yield, dry matter partitioning and physiology of commercial and Erianthus introgressed sugarcane clones under contrasting water regimes. Agric. Water Manag..

[19] Khonghintaisong J., Songsri P., Jongrungklang N. (2021). Understanding growth rate patterns among different drought resistant sugarcane cultivars during plant and ratoon crops encountered water deficit at early growth stage under natural field conditions. Agronomy.

[20] Silva T.R.D., Cazetta J.O., Carlin S.D., Telles B.R. (2017). Drought-induced alterations in the uptake of nitrogen, phosphorus and potassium, and the relation with drought tolerance in sugar cane. Ciênc. Agrotec..

[21] Barbosa A.M., Guidorizi K.A., Catuchi T.A., Marques T.A., Ribeiro R.V., Souza G.M. (2015). Biomass and bioenergy partitioning of sugarcane plants under water deficit. Acta. Physiol. Plant.

[22] Singels A., Donaldson R.A., Smit M.A. (2005). Improving biomass production and partitioning in sugarcane: Theory and practice. Field Crops Res..

[23] Khonghintaisong J., Songsri P., Toomsan B., Jongrungklang N. (2018). Rooting and physiological trait responses to early drought stress of sugarcane cultivars. Sugar Tech..

[24] Reyes J.A.O., Casas D.E., Gandia J.L., Delfin E.F., Gorawala P. (2021). Drought impact on sugarcane production. Agricultural Research Updates.

[25] Leite J.M., Ciampitti I.A., Mariano E., Vieira-Megda M.X., Trivelin P.C. (2016). Nutrient partitioning and stoichiometry in unburnt sugarcane ratoon at varying yield levels. Front. Plant Sci..

[26] Yang Y., Gao S., Jiang Y., Lin Z., Luo J., Li M., Que Y. (2019). The physiological and agronomic responses to nitrogen dosage in different sugarcane varieties. Front. Plant Sci..

[27] Cakmak I. (2002). Plant nutrition research: Priorities to meet human needs for food in sustainable ways. Plant Soil..

[28] Roy E.D., Willig E., Richards P.D., Martinelli L.A., Vazquez F.F., Pegorini L., Spera S.A., Porder S. (2017). Soil phosphorus sorption capacity after three decades of intensive fertilization in Mato Grosso, Brazil. Agric. Ecosyst. Environ..

[29] Tarumoto M.B., de Campos M., Momesso L., do Nascimento C.A.C., Garcia A., Coscolin R.B.D.S., Garcia A., Coscolin R.B.S., Martello J.M., Crusciol C.A.C. (2022). Carbohydrate partitioning and antioxidant substances synthesis clarify the differences between sugarcane varieties on facing low phosphorus availability. Front. Plant Sci..

[30] da Silva E.C., Nogueira R.J.M.C., da Silva M.A., de Albuquerque M.B. (2011). Drought stress and plant nutrition. Plant Stress.

[31] Cakmak I. (2005). The role of potassium in alleviating detrimental effects of abiotic stresses in plants. Nutr. Soil Sci..

[32] Römheld V., Kirkby E.A. (2010). Research on potassium in agriculture: Needs and prospects. Plant Soil.

[33] Zhao Z., Verburg K., Huth N. (2017). Modelling sugarcane nitrogen uptake patterns to inform design of controlled release fertiliser for synchrony of N supply and demand. Field Crops Res..

[34] Lemoine R., Camera S.L., Atanassova R., Dédaldéchamp F., Allario T., Pourtau N., Bonnemain J.L., Laloi M., Coutos-Thévenot P., Maurousset L. (2013). Source-to-sink transport of sugar and regulation by environmental factors. Front. Plant Sci..

[35] Lattanzi F.A., Schnyder H., Thornton B. (2005). The sources of carbon and nitrogen supplying leaf growth. Assessment of the role of stores with compartmental models. Plant Physiol..

[36] Oliveira L.R.D., Miranda G.V., Delima R.O., Neto R.F., Galvão J.C.C. (2013). Nitrogen uptake and utilization efficiency and enzymatic activity in maize genotypes. Rev. Cienc. Agron..

[37] Shah J.M., Bukhari S.A.H., Zeng J.B., Quan X.Y., Ali E., Muhammad N., Zhang G.P. (2017). Nitrogen (N) metabolism related enzyme activities, cell ultrastructure and nutrient contents as affected by N level and barley genotype. J. Integr. Agric..

[38] Cruzat V., Macedo R.M., Keane K.N., Curi R., Newsholme P. (2018). Glutamine: Metabolism and immune function, supplementation and clinical translation. Nutrients.

[39] Kumar R., Sagar V., Verma V.C., Kumari M., Gujjar R.S., Goswami S.K., Jha S.K., Pandey H., Dubey A.K., Srivastava S. (2023). Drought and salinity stresses induced physio-biochemical changes in sugarcane: An overview of tolerance mechanism and mitigating approaches. Front. Plant Sci..

[40] Bhatt R., Singh J., Laing A.M., Meena R.S., Alsanie W.F., Gaber A., Hossain A. (2021). Potassium and water-deficient conditions influence the growth, yield and quality of ratoon sugarcane (*Saccharum officinarum* L.) in a semi-arid agroecosystem. Agronomy.

[41] Raza S., Saleem M.F., Shah G.M., Jamil M., Khan I.H. (2013). Potassium applied under drought improves physiological and nutrient uptake performances of wheat (*Triticum Aestivun* L.). J. Soil Sci. Plant Nutr..

[42] Wang M., Zheng Q., Shen Q., Guo S. (2013). The critical role of potassium in plant stress response. Int. J. Mol. Sci..

[43] Granato Í.S.C., Bermudez F.P., Reis G.G.D., Dovale J.C., Miranda G.V., Fritsche-Neto R. (2014). Index selection of tropical maize genotypes for nitrogen use efficiency. Bragantia.

[44] Chanaphai P., Jongrungklang N., Puangbut D., Songsri P. (2023). Response of photosynthetic and root traits of sugarcane genotypes under drought and recovery conditions. Sugar Tech..

[45] Zhang H., Kaiuki S., Schroder J.L., Payton M.E., Focht C. (2009). Interlaboratory validation of the Mehlich 3 method for extraction of plant–available phosphorus. J. AOAC Int..

[46] Cheong L.N., Kwong K.N.K., Preez C.D. (2009). Effects of sugar cane (*Saccharum hybrid* sp.) cropping on soil acidity and exchangeable base status in Mauritius. South Afr. J. Plant Soil.

[47] Meyer J., Meyer J.H., Turner P.E., Rein P., Mathias K. (2013). Sugarcane nutrition and fertilization. Good Management Practices for the Cane Industry.

[48] Silva M.D.A., Jifon J.L., Santos C.M.D., Jadoski C.J., Silva J.A.G.D. (2013). Photosynthetic capacity and water use efficiency in sugarcane genotypes subject to water deficit during early growth phase. Braz. Arch. Biol. Technol..

[49] Sáez-Plaza P., Navas M.J., Wybraniec S., Michałowski T., Asuero A.G. (2013). An overview of the Kjeldahl method of nitrogen determination. Part II. Sample preparation, working scale, instrumental finish, and quality control. Crit. Rev. Anal. Chem..

[50] Aboyeji C.M., Adekiya A.O., Dunsin O., Adebiyi O.T.V., Aremu C.O., Olofintoye T.A.J., Ajiboye B.O., Owolabi I.O. (2019). Response of soil chemical properties, performance and quality of sweet potato (*Ipomoea Batatas* L.) to different levels of K fertilizer on a tropical Alfisol. Open. Agric..

[51] Gomez K.A., Gomez A.A. (1984). Statistical Procedures for Agricultural Research.

