# Supplementary material for: Differential Nutrient Use Efficiency and Biomass Partitioning of Interspecific Hybrids and Commercial Sugarcane Genotypes Under Early Drought Stress and Recovery Conditions
Source: Plants (Basel). 2025 Dec 5;14(24):3717. doi: 10.3390/plants14243717 (PMC12736929; doi:10.3390/plants14243717)
Supplement: Supplementary file 1 [file plants-14-03717-s001.zip › plants-3965851-supplementary.pdf]

plants

MDPI

*Article*

# Differential Nutrient Use Efficiency and Biomass Partitioning of Interspecific Hybrids and Commercial Sugarcane Genotypes under Early Drought Stress and Recovery Conditions

## Supplementary Table

**Table S1.** Mean squares from ANOVA for biomass, total nitrogen uptake, nitrogen absorption (NAE), nitrogen utilization efficiency (NUtE), and nitrogen use efficiency (NUE) at 6, 8, and 12 MAT under two water regimes of six sugarcane genotypes.

| SOV | DF | Biomass (ton ha$^{-1}$) | | Total N uptake (kg ha$^{-1}$) | | NAE | | NUtE | | NUE | |
|---|---|---|---|---|---|---|---|---|---|---|---|
| 6 MAT | | | | | | | | | | | |
| Block | 2 | 1.1 | | 51.9 | | 0.001 | | 127 | | 124 | |
| Water regime (W) | 1 | 1630.8 | ** | 13587.8 | * | 0.693 | ** | 42684 | * | 83383 | ** |
| Error (a) | 2 | 3.2 | | 319.1 | | 0.009 | | 1322 | | 117 | |
| Genotype (G) | 5 | 77.9 | ** | 1530.9 | ** | 0.078 | ** | 694 | ns | 3969 | ** |
| W x G | 5 | 7.5 | * | 87.1 | ns | 0.004 | ns | 626 | ns | 382 | ns |
| Error (b) | 20 | 2.5 | | 198.9 | | 0.010 | | 587 | | 293 | |
| 8 MAT | | | | | | | | | | | |
| Block | 2 | 94.5 | | 984.5 | | 0.050 | | 5641 | | 3074 | |
| Water regime (W) | 1 | 2445.3 | * | 20851.4 | ** | 1.064 | ** | 7330 | ns | 125104 | ** |
| Error (a) | 2 | 145.3 | | 106.0 | | 0.006 | | 829 | | 112 | |
| Genotype (G) | 5 | 223.7 | ** | 4876.3 | ** | 0.246 | ** | 1458 | * | 11196 | ** |
| W x G | 5 | 80.7 | ** | 659.7 | ns | 0.033 | ns | 1177 | * | 4088 | ** |
| Error (b) | 20 | 8.5 | | 255.6 | | 0.013 | | 398 | | 702 | |
| 12 MAT | | | | | | | | | | | |
| Block | 2 | 145.1 | | 79.1 | | 0.004 | | 242 | | 467 | |
| Water regime (W) | 1 | 6263.9 | * | 16666.8 | * | 0.862 | * | 28249 | ** | 338728 | ** |
| Error (a) | 2 | 141.9 | | 870.3 | | 0.043 | | 93 | | 4157 | |
| Genotype (G) | 5 | 522.6 | ** | 6332.2 | ** | 0.323 | ** | 11656 | ** | 29189 | ** |
| W x G | 5 | 21.0 | ns | 2029.5 | ** | 0.104 | ** | 5340 | ** | 1453 | ns |
| Error (b) | 20 | 47.2 | | 397.3 | | 0.020 | | 1314 | | 1840 | |

ns = non significance, * Significant at p = 0.05, ** significant at p = 0.01. DF: Degree of freedom, SOV: Source of variation

 https://doi.org/10.3390/xxxxx

**Table S2.** Mean squares from ANOVA for total phosphorus uptake, phosphorus absorption (PAE), phosphorus utilization efficiency (PUtE), phosphorus efficiency (PUE), potassium in the plant, potassium absorption (KAE), potassium utilization efficiency (KUtE), and potassium use efficiency (PUE) at 6, 8, and 12 MAT under two water regimes of six sugarcane genotypes.

| SOV | DF | Total P uptake (kg ha$^{-1}$) | | PAE | | PUtE | | PUE | | K in plant (kg ha$^{-1}$) | | KAE | | KUtE | | KUE | |
|---|---|---|---|---|---|---|---|---|---|---|---|---|---|---|---|---|---|
| 6 MAT | | | | | | | | | | | | | | | | | |
| Block | 2 | 41.4 | | 0.16 | | 66115 | | 4257 | | 502 | | 0.03 | | 0.8 | | 58 | |
| Water regime (W) | 1 | 1531.4 | ** | 5.88 | ** | 35974 | ns | 6279760 | ** | 236407 | ** | 12.36 | ** | 94.8 | ns | 85351 | ** |
| Error (a) | 2 | 22.7 | | 0.09 | | 19853 | | 11843 | | 102 | | 0.01 | | 25.5 | | 161 | |
| Genotype (G) | 5 | 107.8 | ** | 0.41 | ** | 48142 | ns | 298913 | ** | 13343 | ** | 0.70 | ** | 219.3 | ** | 4063 | ** |
| W x G | 5 | 2.0 | ns | 0.01 | ns | 8878 | ns | 28760 | * | 1029 | ns | 0.05 | ns | 188.7 | ** | 391 | * |
| Error (b) | 20 | 9.2 | | 0.04 | | 21671 | | 9875 | | 807 | | 0.04 | | 37.2 | | 134 | |
| 8 MAT | | | | | | | | | | | | | | | | | |
| Block | 2 | 102.4 | | 0.40 | | 140557 | | 48958 | | 923 | | 0.05 | | 236.3 | | 799 | |
| Water regime (W) | 1 | 2106.8 | * | 8.14 | * | 8789 | ns | 1 x$10^7$ | ** | 131951 | ** | 6.94 | ** | 2069.8 | * | 127710 | ** |
| Error (a) | 2 | 66.0 | | 0.25 | | 51006 | | 93774.6 | | 903 | | 0.05 | | 35.8 | | 30 | |
| Genotype (G) | 5 | 115.5 | ** | 0.44 | ** | 134924 | ** | 893344 | ** | 29549 | ** | 1.54 | ** | 186.9 | ns | 11689 | ** |
| W x G | 5 | 23.9 | ns | 0.09 | ns | 45533 | * | 338742 | ns | 12860 | ** | 0.67 | ** | 264.5 | ns | 4208 | ** |
| Error (b) | 20 | 26.4 | | 0.10 | | 15628 | | 218211 | | 1682 | | 0.09 | | 246.5 | | 364 | |
| 12 MAT | | | | | | | | | | | | | | | | | |
| Block | 2 | 82.9 | | 0.32 | | 54355 | | 22192 | | 1369 | | 0.07 | | 32.2 | | 2302 | |
| Water regime (W) | 1 | 3776.7 | * | 14.53 | * | 550 | ns | 3x$10^7$ | ** | 424283 | ** | 22.17 | ** | 420.6 | ns | 327470 | ** |
| Error (a) | 2 | 64.6 | | 0.25 | | 22207 | | 184933 | | 1118 | | 0.06 | | 34.0 | | 425 | |
| Genotype (G) | 5 | 755.7 | ** | 2.91 | ** | 327953 | ** | 2331206 | ** | 144368 | ** | 7.55 | ** | 2303.5 | ** | 27319 | ** |
| W x G | 5 | 69.9 | ns | 0.27 | ns | 38626 | ns | 135784 | ns | 10473 | * | 0.55 | * | 1334.4 | ** | 1099 | ns |
| Error (b) | 20 | 51.6 | | 0.20 | | 42942 | | 190896 | | 3411 | | 0.18 | | 299.5 | | 782 | |

ns = non significance, * Significant at p = 0.05, ** significant at p = 0.01. DF: Degree of freedom, SOV: Source of variation
